# Genetic Contribution of Variants near *SORT1* and *APOE* on LDL Cholesterol Independent of Obesity in Children

**DOI:** 10.1371/journal.pone.0138064

**Published:** 2015-09-16

**Authors:** Clara Breitling, Arnd Gross, Petra Büttner, Sebastian Weise, Dorit Schleinitz, Wieland Kiess, Markus Scholz, Peter Kovacs, Antje Körner

**Affiliations:** 1 Center for Pediatric Research (CPL), University Hospital for Children and Adolescents Leipzig, Dept. of Women´s & Child Health, University of Leipzig, Leipzig, Germany; 2 Institute for Medical Informatics, Statistics and Epidemiology (IMISE), University of Leipzig, Leipzig, Germany; 3 LIFE-Leipzig Research Center for Civilization Diseases, University of Leipzig, Leipzig, Germany; 4 Integrated Research and Treatment Center Adiposity Diseases (IFB), Medical Faculty, University of Leipzig, Leipzig, Germany; University of Leipzig, GERMANY

## Abstract

**Objective:**

To assess potential effects of variants in six lipid modulating genes (*SORT1*, *HMGCR*, *MLXIPL*, *FADS2*, *APOE* and *MAFB*) on early development of dyslipidemia independent of the degree of obesity in children, we investigated their association with total (TC), low density lipoprotein (LDL-C), high density lipoprotein (HDL-C) cholesterol and triglyceride (TG) levels in 594 children. Furthermore, we evaluated the expression profile of the candidate genes during human adipocyte differentiation.

**Results:**

Expression of selected genes increased 10^1^ to >10^4^ fold during human adipocyte differentiation, suggesting a potential link with adipogenesis. In genetic association studies adjusted for age, BMI SDS and sex, we identified significant associations for rs599839 near *SORT1* with TC and LDL-C and for rs4420638 near *APOE* with TC and LDL-C. We performed Bayesian modelling of the combined lipid phenotype of HDL-C, LDL-C and TG to identify potentially causal polygenic effects on this multi-dimensional phenotype and considering obesity, age and sex as a-priori modulating factors. This analysis confirmed that rs599839 and rs4420638 affect LDL-C.

**Conclusion:**

We show that lipid modulating genes are dynamically regulated during adipogenesis and that variants near *SORT1* and *APOE* influence lipid levels independent of obesity in children. Bayesian modelling suggests causal effects of these variants.

## Introduction

Alterations in blood lipid phenotypes culminating in dyslipidemia are important risk factors for the development of cardiovascular disease [1]. Elevated blood low-density lipoprotein cholesterol (LDL-C) and triglycerides (TG) are strongly related to the likelihood of existing or future coronary heart disease [2, 3], whereas elevated blood high-density lipoprotein cholesterol (HDL-C) has a protective effect [4]. The most common cause of dyslipidemia is obesity [5]. However, a relevant proportion of patients with elevated blood lipid levels does not show an abnormal BMI [6].

Meta-analyses of genome-wide association (GWA) studies revealed many genetic loci influencing blood lipid levels underlying the polygenic cause of dyslipidemia and thereby identified suspected as well as unsuspected new candidate genes [7–10]. However, these meta-analyses concern adult cohorts. So far, there are only very few data on selected genes associated with altered blood lipid phenotypes in children and adolescents [11–14].

Investigation of childhood cohorts has several advantages though. They are much less biased by chronic disease and treatments but already show considerable heterogeneity regarding blood lipid levels. Also, considering future prediction of developing dyslipidemia, it is important to assess whether associations between genetic variants and blood lipid phenotypes observed in adults are already evident in children and adolescents [15]. Due to the lower influence of co-morbidities and other life-style related factors, we suppose that primary genetic effects are stronger in children than in adults. Thus, we hypothesize that we can detect at least some of the variants even with the lower number of individuals available for childhood cohorts.

In the present study, we aimed at assessing associations of six variants with lipid traits in a sample of mainly obese children. Selected variants are located in or near the genes *SORT1* (sortilin 1), *HMGCR* (3-hydroxy-3-methylglutaryl-Coenzyme A reductase), *MLXIPL* (*MLX interacting protein)*, *FADS2* (fatty acid desaturase 2), *APOE* (apolipoprotein E) and *MAFB* (V-maf musculoaponeurotic fibrosarcoma oncogene homolog B) for which high effect-sizes regarding lipid phenotypes were reported. Going beyond classical association analysis, we additionally performed a Bayesian modelling approach to identify unconfounded relationships between genetic and non-genetic covariables and lipid phenotypes. Considering that obesity is a risk factor for dyslipidemia *per se* and that adipose tissue is an important tissue for lipid metabolism, we also assessed a potential relationship of the candidate gene expression for adipogenesis by studying time-series of gene-expression during human adipocyte differentiation.

## Methods

### Selection of Candidate Genes and Variants

Genes were selected according to evidence of genotype-phenotype-associations established in meta-analyses of adult cohorts [8–10, 16, 17]. We prioritized genetic variants by applying a score integrating (i) GWAS for lipid genes and obesity (p-value), (ii) gene expression data from adipocytes, (iii) minor allele frequency and effect size, (iv) verification in replication analyses,. Based on these criteria, we selected six variants rs6102059 (*MAFB*), rs4420638 (*APOE*), rs599839 (*SORT1*), rs3846663 (*HMGCR*), rs174570 (*FADS2*) and rs3812316 (*MLXIPL*). We excluded well-known SNPs in genes like LDL-receptor because we were interested in new candidate genes influencing blood lipid levels.

Cis-eQTL effects of SNPs in linkage disequilibrium with our variants were observed for *SORT1* [18–20], *HMGCR* [21] and *FADS2* [21–23].

### Sample

A total of 683 children were recruited from the Leipzig area via our out-patient obesity clinic. We applied German reference data for the calculation of the SDS as suggested by the National German Guidelines for Pediatric Obesity [24]. Obesity was defined as BMI SDS >1.88 corresponding to the 97^th^ percentile.

White children were phenotyped by age, sex, height, weight, pubertal state, laboratory parameters and other clinical characteristics. Assessment of pubertal stage was performed by clinical examination according to Tanner [25, 26]. Blood lipid levels (triglycerides, total cholesterol, HDL and LDL) were determined with direct enzymatic colorimetric assays by the certified laboratory at the Institute of Laboratory Medicine, Clinical Chemistry and Molecular Diagnosis at the University of Leipzig. Written informed consent was obtained from all parents and from participants ≥12 years of age. This study has been approved by the ethics committee of the University of Leipzig and has been conducted according to the principles expressed in the Declaration of Helsinki (October 2000).

We excluded children with chronic inflammatory diseases, metabolic diseases, genetic disorders and diseases that required medication influencing lipid metabolism (N = 89).

For the remaining 594 children, data on glucose metabolism and lipid phenotypes (TC, LDL-C, HDL-C, TG) were available. Anthropometric and metabolic characterisation of included samples is presented in Table 1.

**Table 1:** Anthropometric and metabolic characterization of study samples.

Sex (male / female)	277 / 317
n (non-obese / obese)	122 / 472
BMI SDS	2.39 (0.85)
Age (years)	11.67 (5.23)
Total Cholesterol (mmol/L)	4.08 (0.99)
HDL-C (mmol/L)	1.22 (0.37)
LDL-C (mmol/L)	2.46 (0.89)
Triglyceride (mmol/L)	0.99 (0.70)

Quantitative variables are presented as median (interquartile range). Obesity is defined as BMI SDS>1.88.

### Gene expression analysis during human adipocyte differentiation

Gene expression profiles of selected genes were determined via qRT-PCR for human preadipocyte SGBS (Simpson-Golabi-Behmel syndrome) cells during differentiation into mature adipocytes. Adipocyte differentiation was induced as described previously [27].

RNA extraction was performed using the RNeasy MiniKit (Qiagen, Hilden, Germany) including DNase digestion according to the manufacturer’s instructions. Reverse transcription of 50 ng/μl RNA was carried out using the M-MLV Reverse Transcriptase Kit (Invitrogen, Karlsruhe, Germany) with random hexamer [p(dN)_6_] primers (Roche, Basel, Switzerland). Primer sequences are provided in S1 Table.

Experiments were performed in three distinct experiments each in triplicates. Target gene-expression was normalized to the averaged expression of three housekeeping genes *TBP*, *HPRT* and *USF2*.

### DNA-Isolation and genotyping

Fasting venous EDTA blood samples were stored at -80°C. After washing with phosphate buffered saline, erythrocyte depletion by NH_4_-lysis, and centrifugation, we extracted DNA using QIAmp DNA Blood MiniKit (Qiagen) according to the manufacturer’s manual.

Genotyping probes and primers were obtained from Applied Biosystems (Darmstadt, Germany). Primer sequences are listed in S2 Table. We used qPCR MasterMix Kit for probe Assay and Low Rox Plus (Eurogentec, Köln, Germany) for genotyping according to the manufacturers’ manuals. Genotyping was performed on ABI Prism 7500 sequence detector (Applied Biosystems, Lincoln, USA). At least 5% of all samples were re-assessed on a different plate with concordance rate of 100%. Genotype frequencies of all SNPs were consistent with Hardy-Weinberg equilibrium. SNP characteristics are presented in S3 Table.

### Classical statistical analysis

In classical analysis of genetic data, every combination of a single SNP and a single phenotype is tested for association. Prior to analysis, lipid phenotypes TC, LDL-C, HDL-C and TG were log transformed to approximate normal distribution. Continuous phenotypes TC, LDL-C, HDL-C, TG, age and BMI SDS were standardised to zero mean and unit variance before analysis in order to obtain dimensionless effect estimates which are better comparable between different predictors and studies.

SNP associations with BMI SDS were tested with a linear model assuming three different genetic models, an additive effect of both alleles, and a dominant and recessive effect of the major allele, respectively. All models were adjusted for age and sex. Similarly, lipid SNP associations were tested with a linear model and three different genetic models. All models were adjusted for age, BMI SDS and sex. Adjustment for pubertal state instead of age is also reasonable. Due to the high correlation of age and pubertal state (Spearman r = 0.84), the genetic results are essentially the same (not shown). Also, pubertal stage is assessed by two parameters (pubic hair and breast development or testicular volume), which are not necessarily coherent. Furthermore, pubertal timing differs between boys and girls. Since dyslipidemia would be more related to age as an indicator of duration of obesity and dyslipidemia it is not necessarily influenced by pubertal development per se, this was another reason to adjust for age. We, therefore, decided to use the continuous and less ambiguously measurable trait age instead of pubertal state.

Since we tested five phenotypes, six SNPs and three genetic models, it is necessary to correct for multiple testing. However, due to multiple correlations between phenotypes and effects of genetic models, it was necessary to simulate the null-distribution. In our situation, a significance level of 6.7x10^-4^ controls the family-wise error rate at 5% and was therefore used to correct for multiple testing in our study.

Statistical analysis was performed using R 2.10.1.

### Bayesian Model Analysis

The major drawbacks of the classical analysis mentioned above are the large number of tests to be performed due to the large number of possible combinations of SNPs and phenotypes and the assumption of a specific model of genetic and non-genetic effects. To overcome these limitations, we performed Bayesian model analysis in addition to our classical association analysis. By this approach, we can estimate plausibilities of different models and corresponding effect sizes. Bayesian modelling also allows some kind of causal inference by analysing all lipid phenotypes and possible influencing factors in parallel considering their overall correlation structure. To some extent, this avoids spurious associations.

The method is well conceived with application in analysing complex genotype-phenotype associations in medical research [28–30]. Additional insights can be derived from the modelling such as probability of different genetic risk models and estimates of unconfounded effects considering all dependencies between variables of interest. It also circumvents the above mentioned issue of multiple-testing and the uncertainty regarding the model of inheritance.

Similar to the univariate analysis, transformed and standardised data were used. Lipid phenotypes were modelled with the Bayesian variable selection approach described in [29, 31] using the reversible jump interface of WinBUGS (Version 1.4.3). Since correlation of TC and LDL-C is very high (r = 0.91) we studied models of the (three-dimensional) lipid phenotype HDL-C, LDL-C and TG. We aimed to identify the most plausible sets of co-variables having a direct influence on each lipid phenotype accounting for correlations between them.

In our analysis, the set of co-variables consists of age, BMI SDS, sex and a recessive and a dominant part for each of the six SNPs defined by indicator variables “genotype” = 0 and “genotype” = 2 respectively. If both indicator variables are included, different levels of co-dominance can be expressed by corresponding effect estimates. Hence, 15 co-variables were available for selection for each of the 3 lipid phenotypes.

Each different subset of these co-variables forms a model. Prior to analysis, one assumes that all models are equally likely. We calculated Bayesian posterior probabilities representing the plausibilities of the models given our data. Details of Bayesian modelling and fitting can be found in S1 Methods.

Bayes factors [32] are used to interpret model results. Calculation of Bayes factors is explained in the S2 Methods. A usual convention is that a Bayes factor of 1 to 3.2 is judged as “not worth more than a bare mention”, a factor of 3.2 to 10 as “substantial”, a factor of 10 to 100 as “strong” and a factor greater than 100 as “decisive” evidence for a model or effect [33]. Conversely, reciprocal values represent counter-evidence for a model. Rather than deciding whether a certain covariable has an effect or not (i.e. is in the model or not), we calculate corresponding inclusion probabilities, which can be interpreted as plausibilities regarding the impact of the covariable on the phenotype considered. Effect estimates of co-variables can be determined in the Bayesian context by averaging over all models containing this co-variable (Bayesian model averaging) weighted by the plausibility of the model. Results can be considered as analogons to Beta-coefficients of classical linear regression analysis.

## Results

### Classical genotype-phenotype analyses for BMI SDS and lipid phenotypes

There was no significant association between BMI SDS and any of the selected SNPs indicating that the variants are not related to the degree of obesity in our data. Results for the additive model are presented in Table 2. Results for all three genetic models are given in S1 Results.

**Table 2:** Association of genotypes with BMI SDS and lipid phenotypes.

Phenotype	Variant	N	Beta	SE	CI	*p*-value
BMI SDS	rs599839	576	-0.087	0.066	[-0.217;0.044]	0.193
BMI SDS	rs3846663	572	0.076	0.061	[-0.045;0.196]	0.219
BMI SDS	rs3812316	564	-0.126	0.095	[-0.312;0.06]	0.184
BMI SDS	rs174570	578	0.176	0.09	[-0.001;0.353]	0.052
BMI SDS	rs4420638	584	-0.004	0.079	[-0.16;0.152]	0.958
BMI SDS	rs6102059	575	0.062	0.065	[-0.065;0.19]	0.335
**TC**	**rs599839**	**576**	**-0.257**	**0.067**	**[-0.389;-0.125]**	**1.50x10** ^**-4**^
TC	rs3846663	572	0.141	0.062	[0.019;0.263]	0.024
TC	rs3812316	564	-0.022	0.094	[-0.207;0.162]	0.812
TC	rs174570	578	-0.04	0.092	[-0.22;0.14]	0.662
**TC**	**rs4420638**	**584**	**0.336**	**0.079**	**[0.181;0.491]**	**2.45x10** ^**-5**^
TC	rs6102059	575	0.019	0.065	[-0.109;0.146]	0.775
HDL-C	rs599839	576	0.077	0.067	[-0.054;0.207]	0.25
HDL-C	rs3846663	572	0.098	0.061	[-0.021;0.217]	0.106
HDL-C	rs3812316	564	0.129	0.093	[-0.054;0.312]	0.168
HDL-C	rs174570	578	-0.078	0.09	[-0.254;0.098]	0.387
HDL-C	rs4420638	584	-0.13	0.078	[-0.283;0.024]	0.098
HDL-C	rs6102059	575	0.038	0.064	[-0.087;0.164]	0.547
**LDL-C**	**rs599839**	**576**	**-0.3**	**0.067**	**[-0.431;-0.168]**	**8.82x10** ^**-6**^
LDL-C	rs3846663	572	0.12	0.062	[-0.002;0.241]	0.054
LDL-C	rs3812316	564	-0.043	0.094	[-0.226;0.141]	0.649
LDL-C	rs174570	578	0.021	0.091	[-0.158;0.2]	0.817
**LDL-C**	**rs4420638**	**584**	**0.382**	**0.078**	**[0.228;0.536]**	**1.38x10** ^**-6**^
LDL-C	rs6102059	575	0.018	0.065	[-0.109;0.145]	0.781
TG	rs599839	576	-0.114	0.065	[-0.241;0.014]	0.081
TG	rs3846663	572	-0.006	0.059	[-0.123;0.11]	0.913
TG	rs3812316	564	-0.134	0.088	[-0.307;0.038]	0.127
TG	rs174570	578	0.137	0.087	[-0.034;0.307]	0.116
TG	rs4420638	584	0.135	0.076	[-0.014;0.285]	0.076
TG	rs6102059	575	0.075	0.062	[-0.046;0.197]	0.225

We present numbers of cases available for the corresponding analysis (N), beta-coefficients, their standard errors (SE), 95% confidence intervals (CI) and uncorrected *p*-values. Since standardized values were analysed, beta-coefficients and standard errors have unit 1. BMI SDS was analysed with the additive model adjusted for age and sex. Lipid phenotypes were logarithmized and analysed with the additive model adjusted for age, sex and BMI SDS. Associations significant after correction for multiple testing (see methods section) are printed in bold.

Next, we analysed the association between genotypes and lipid phenotypes. We found significant associations with lipid phenotypes for *SORT1* rs599839 with TC (*p* = 1.50x10^-4^, β = -0.257) and LDL-C (*p* = 8.82x10^-6^, β = -0.3) as well as for *APOE* rs4420638 with TC (*p* = 2.45x10^-5^, β = 0.336) and LDL-C (*p* = 1.38x10^-6^, β = 0.382), whereas the variants were not associated with other lipid phenotypes (Table 2). No additional associations were found when investigating alternative models of inheritance (see S1 Results).

### Bayesian model analysis

**Fig 1:**
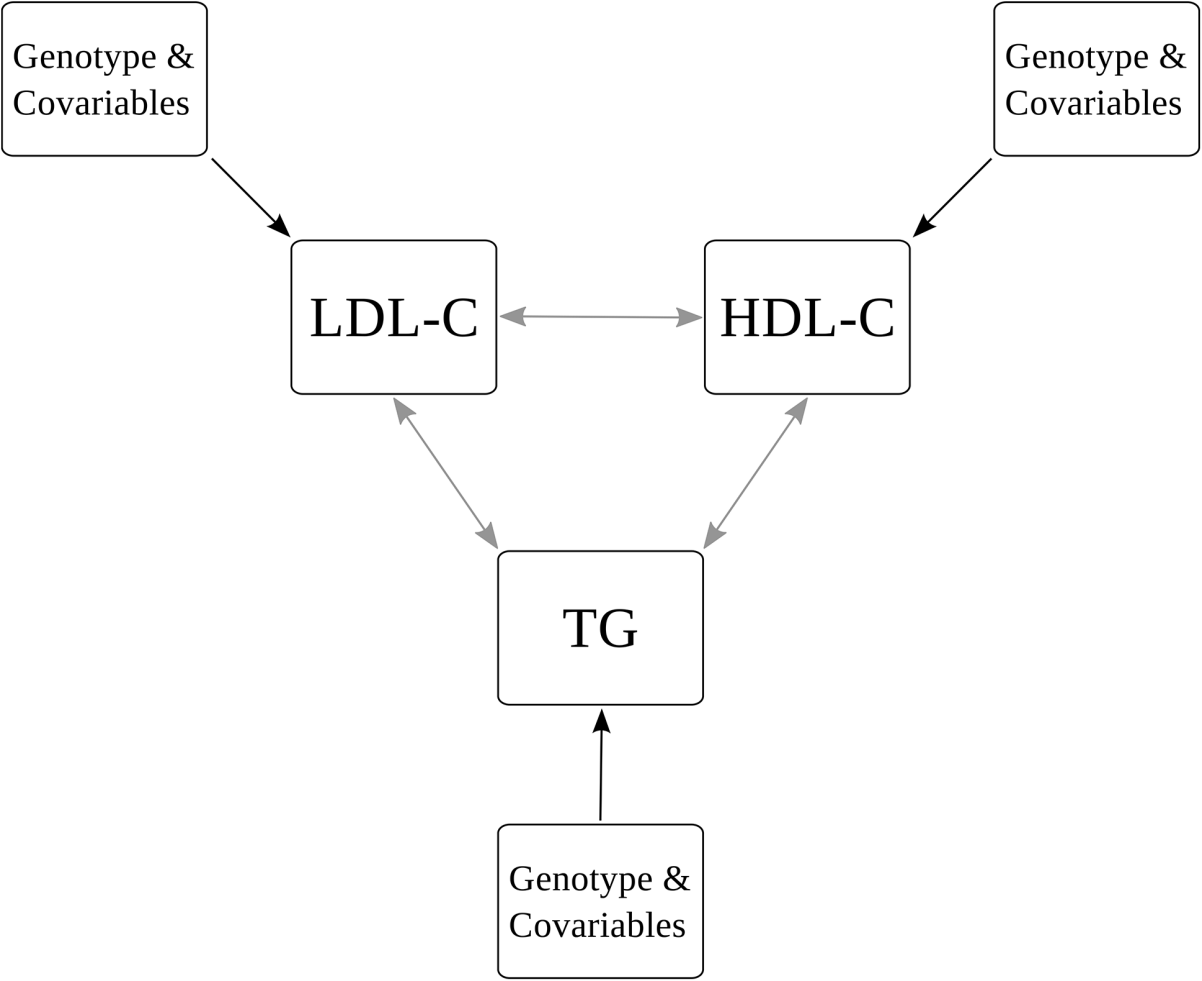
Bayesian Model. We present the structure of the Bayesian model analysed. Black arrows represent possible impacts of considered covariables (SNPs, age, BMI SDS, sex) on the distribution means of lipid phenotypes. Grey arrows refer to the covariance between the lipids which is accounted for in the model.

We performed Bayesian modelling of the multi-phenotype of HDL-C, LDL-C and TG. TC was not included into the model due to its strong correlation with LDL-C. Analysed relations are illustrated in Fig 1. In the following, we present the most plausible models of each lipid phenotype accounting for their pairwise correlations. The corresponding WinBUGS Model is given in detail in S3 Methods. Most probable models in decreasing order of plausibility and corresponding Bayes factors are shown in Table 3. The lists are truncated when the cumulative probability of the models exceeds 95%, i.e. all other models are less plausible according to our data. Both top models of HDL-C contain no genetic factors but age and BMI SDS as co-variables. The third most probable model includes the dominant part of rs4420638 (*APOE*).

**Table 3:** Results of Bayesian model analysis.

Lipid	Model	Probability	Bayes factor
HDL-C	BMI SDS	91.89	371265
HDL-C	age, BMI SDS	3.08	1041
HDL-C	rs4420638_dom_, BMI SDS	0.99	329
LDL-C	rs599839_rec_, rs4420638_rec_	53.49	37691
LDL-C	rs599839_rec_, rs4420638_rec_, BMI SDS	22.88	9720
LDL-C	rs4420638_rec_	7.65	2714
LDL-C	rs4420638_rec_, BMI SDS	4.62	1586
LDL-C	rs599839_rec_	2.54	855
LDL-C	rs599839_dom_, rs4420638_rec_	1.03	340
LDL-C	rs599839_rec_, rs4420638_rec_, age	0.8	266
LDL-C	rs599839_rec_, rs4420638_rec_, rs6102059_dom_	0.77	254
LDL-C	rs599839_rec_, BMI SDS	0.74	244
LDL-C	null	0.56	186
TG	age, BMI SDS	90.47	311171
TG	rs3812316_dom_, age, BMI SDS	3.66	1247
TG	BMI SDS	2.55	856

Possible models of HDL-C, LDL-C, TG can contain up to 15 covariables (age, sex, BMI SDS, dominant and recessive effect of six SNPs). We present most probable models, corresponding posterior probabilities and Bayes factors. Models are ranked according to their plausibility. A cumulative probability of 95% served as cut-off for model presentation.

The top models of TG contain age and BMI SDS, too. Additionally, the second best model includes the dominant part of rs3812316 (*MLXIPL*).

Various different models are plausible for LDL-C: The recessive parts of SNP rs599839 (*SORT1*) and rs4420638 (*APOE*) and BMI SDS contribute to the top 5 models of LDL-C. In less probable models for LDL-C, combinations of the recessive and dominant parts of rs599839 and rs4420638, age and BMI SDS occur. Further, the dominant part of rs6102059 (*MAFB*) is included once.

**Fig 2:**
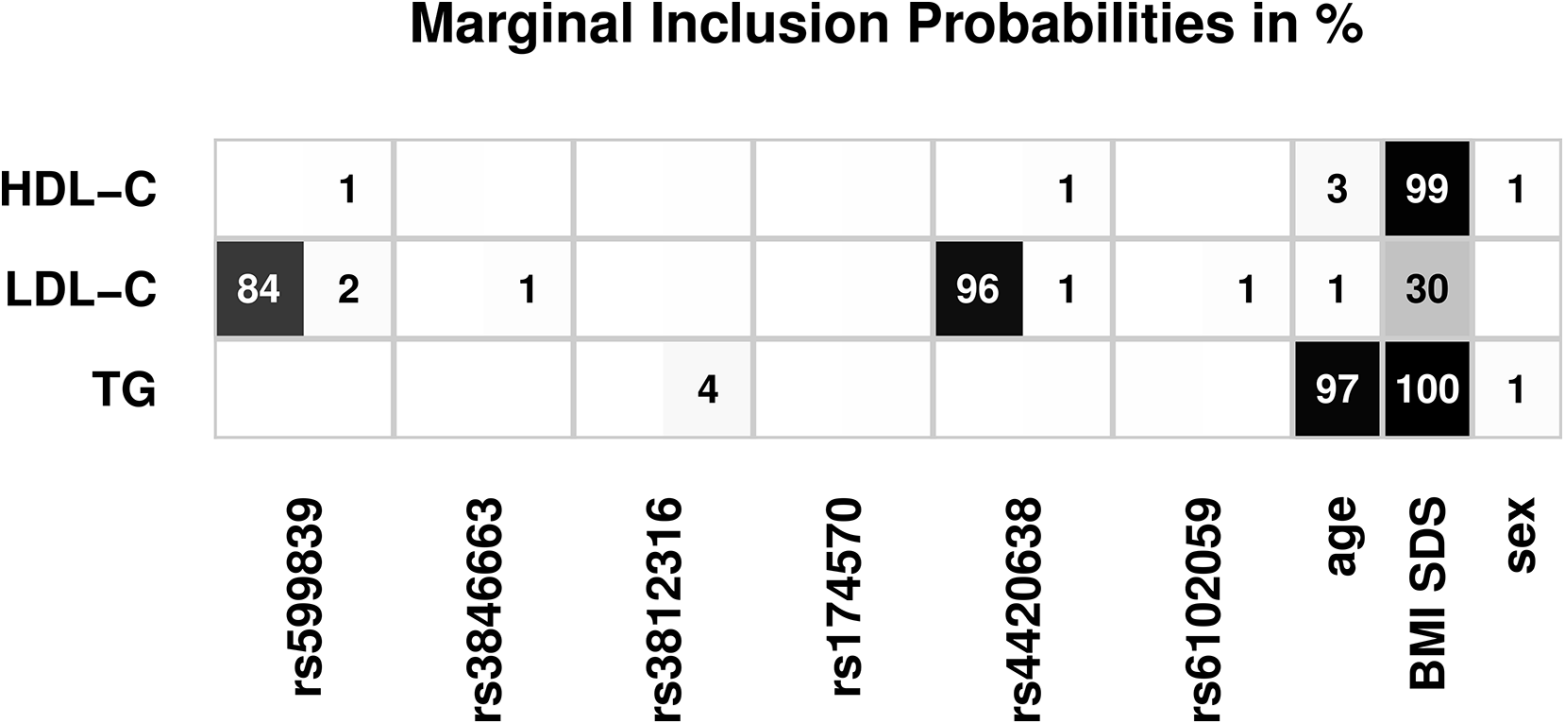
Inclusion probabilities of covariables for each lipid phenotype. For each SNP, results are given for the recessive (first number) and dominant part (second number). Results for inclusion probabilities are rounded to integers of percentage. Effect estimates are illustrated by the shade of grey as indicated. Results rounded to zero are omitted. Results for the lipid phenotypes LDL-C, HDL-C and TG are presented. TC is omitted due to high correlation with LDL-C.

The impact of each co-variable independent of a certain model can be assessed by interpreting the inclusion probabilities for co-variables (Fig 2). In addition to the apparent and expected impact of BMI SDS, rs599839 (*SORT1*) and rs4420638 (*APOE*) have a high certainty of affecting LDL-C independent of the degree of obesity. Conversely, the following effects cannot be ruled out (i.e. no decisive evidence against the effect was found): rs4420638 (*APOE*) on HDL-C, rs3846663 (*HMGCR*) and rs6102059 (*MAFB*) on LDL-C, rs3812316 (*MLXIPL*) on TG.

Effect estimates of co-variables with inclusion probability greater than 0.5% are listed in Table 4. Estimates and standard deviations are averaged over all models, where the respective co-variable is included (Bayesian model averaging). In comparison to classical analysis, the majority of standard deviations of estimates are smaller demonstrating higher power of the Bayesian model approach (S1 Fig).

**Table 4:** Inclusion probabilities of covariables and Bayesian effect sizes.

Lipid	Variant	Probability	Estimate	SD
HDL-C	rs599839_dom_	0.6	0.253	0.178
HDL-C	rs4420638_dom_	1.03	-0.415	0.25
HDL-C	age	3.22	-0.127	0.041
HDL-C	BMI SDS	99.34	-0.21	0.041
HDL-C	sex	0.53	-0.147	0.072
LDL-C	rs599839_rec_	84.3	0.32	0.077
LDL-C	rs599839_dom_	2.2	-0.415	0.171
LDL-C	rs3846663_dom_	1.16	0.258	0.114
LDL-C	rs4420638_rec_	95.57	-0.365	0.081
LDL-C	rs4420638_dom_	0.58	0.347	0.239
LDL-C	rs6102059_dom_	1.23	0.276	0.134
LDL-C	age	1.18	-0.12	0.042
LDL-C	BMI SDS	30	0.146	0.04
TG	rs3812316_dom_	3.81	-0.757	0.346
TG	age	97.28	0.172	0.035
TG	BMI SDS	99.98	0.255	0.044
TG	sex	1.45	-0.166	0.061

We present probabilities for inclusion of specified covariables and resulting effect sizes and corresponding standard deviations (SD) averaged over all models containing the covariable. Only covariables with an inclusion probability greater than 0.5% are shown.

The estimated covariance of the model is shown in S2 Results. Results of the combined model of TC, HDL-C, TG are similar to those of the model of LDL-C, HDL-C, TG considered here (data not shown).

Polygenic effects for LDL-C are illustrated in S2 Fig.

### Gene-expression analysis in human adipocyte precursors

**Fig 3:**
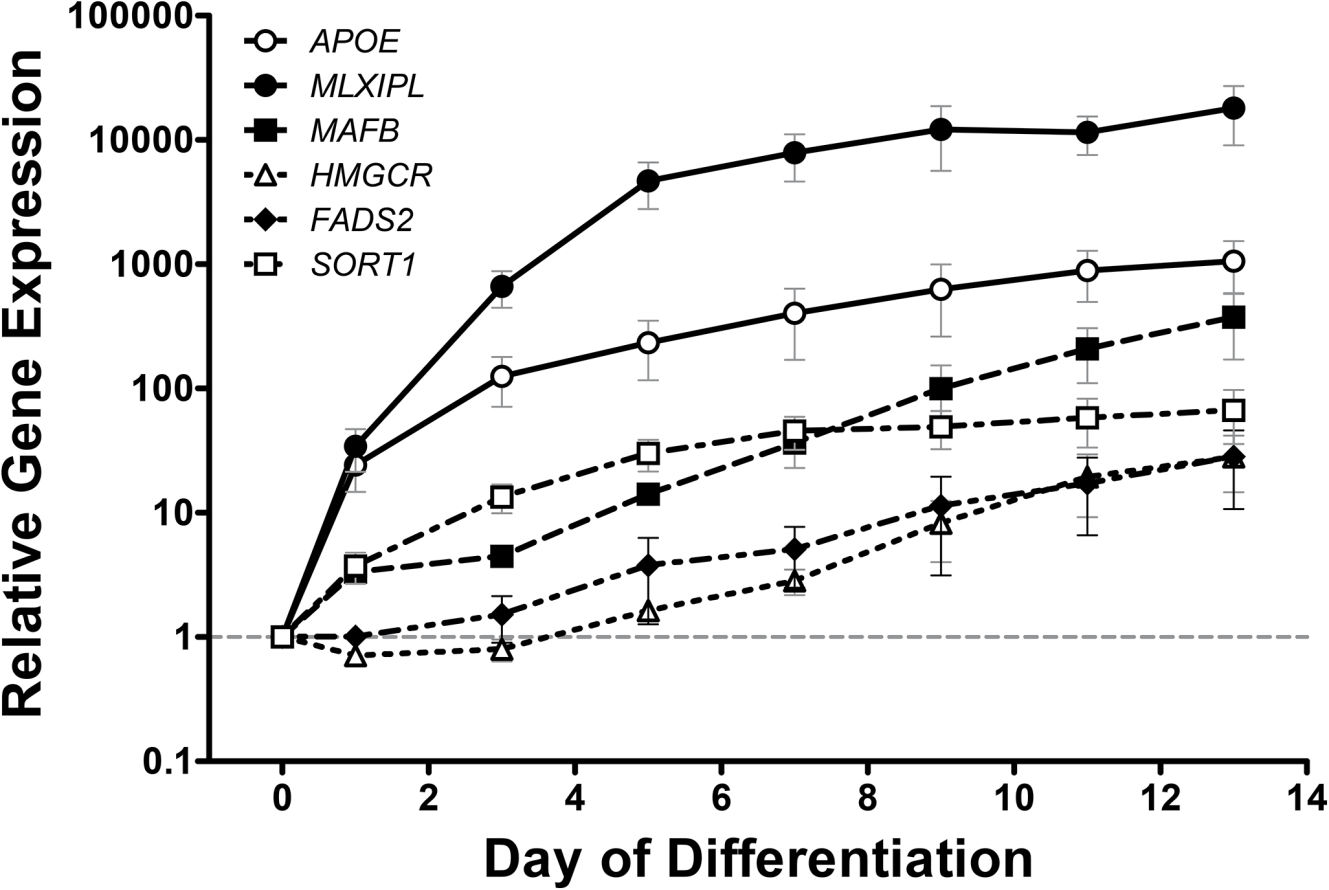
mRNA expression profiles of target genes during human adipogenesis. Fold change of gene expression for *SORT1*, *HMGCR*, *MLXIPL*, *FADS2*, *APOE* and *MAFB* mRNA during human adipocyte differentiation of SGBS cells. Data shown are averaged over 3 independent experiments, each performed in triplicates and results are given in mean±SEM. For all candidates, *p*<0.001 was achieved by one-way ANOVA test with Dunnet´s posthoc test.

We measured gene expression of *SORT1*, *HMGCR*, *MLXIPL*, *FADS2*, *APOE* and *MAFB* during differentiation of human preadipocytes into adipocytes to assess a potential physiological relevance in lipid metabolism. We observed an up-regulation of these genes by magnitudes of 10 to 10^4^ (Fig 3).

## Discussion

In this study, we aimed to assess the relevance of SNPs showing associations with lipid phenotypes from adults in a childhood sample which is less prone to confounding factors such as medication and co-morbidities and has shorter exposure to endogenous and exogenous factors.

Considering the strong impact of obesity and hence adipose tissue on circulating lipid phenotypes, we were also interested whether the candidate genes are dynamically regulated during adipogenesis. We have previously shown that genetic candidates from GWAS for obesity traits may have a functional role in human adipogenesis [27]. All selected genes from this study were expressed in adipocytes and showed considerable up-regulations during human adipocyte differentiation up to 10,000 fold. This has not been shown for these genes before. Even though this dynamic regulation during adipogenesis does not directly imply a functional relationship, this finding merits further investigation in mechanistic studies. We evaluated the dynamic regulation of candidate gene expression in SGBS preadipocytes, so far the only established model of human preadipocyte differentiation [34], which is widely applied in adipogenesis research. It has been shown that biology and molecular markers are comparable to primary human adipocyte differentiation and circumvents potential bias by patient heterogeneity due to age, risk factors, morbidities, treatment etc.

Considering the strong dependence of lipid levels on obesity, the observed regulations may affect serum lipid phenotypes and may explain SNP associations. We, therefore, verified that the six variants considered were not associated with the degree of obesity in the children prior to evaluation of associations with lipid traits.

Of the six selected SNPs located in or near the genes *FADS2* (rs174570), *MAFB* (rs6102059), *HMGCR* (rs3846663), *MLXIPL* (rs3812316), *APOE/C1/C4/C2* (rs4420638) and *CELSR2/PSRC1/SORT1* (rs599839), we observed a strong impact of rs599839 (*SORT1*) and rs4420638 (*APOE*) on circulating LDL-C levels independent of the degree of obesity in conventional linear regression analyses adjusting for age, sex and BMI SDS. This was confirmed by our Bayesian model analysis suggesting causality of these two variants on LDL-C. Bayesian analysis also revealed that effects of rs4420638 (*APOE*) on HDL-C, rs3846663 (*HMGCR*) and rs6102059 (*MAFB*) on LDL-C as well as rs3812316 (*MLXIPL*) on TG cannot be ruled out. Still, these variants should be considered as candidates requiring further investigations.

The *APOE*-SNP rs4420638 is located on chromosome 19 in a cluster with *APOC1*, *APOC4* and *APOC2*. The SNP rs599839 is located on chromosome 1, close to the genes *CELSR2*, *PSRC1* and *SORT1*. Multiple other studies investigated SNPs in or near the *APOE* and *SORT1* genes. Rs4420638 and rs599839 showed replicable associations with lipid levels (mostly LDL-C) in Caucasian and non-Caucasian population cohorts and meta-analyses [7, 9, 15, 35]. The non-Caucasian cohorts displayed lower significance regarding all SNP-lipid-associations, most likely due to lower case numbers (N ranges from 2,532 to 9,328) [15]. In a small sample, Klein et al. observed effects of rs646776, a proxy of rs599839, only in males [36]. Sex-stratified analysis of our data reveals a significant effect for both sexes. Beta estimator of females is slightly lower than that for males (Females: *p* = 6.5x10^-4^, β = -0.28, Males: *p* = 4.5x10^-3^, β = -0.32).

Associations of the other variants/candidates could not be confirmed in our sample. Correlations for rs174570 (*FADS2*) with TC, LDL-C and HDL-C were observed in a meta-analysis of 16 European studies [10], although others did not confirm this in Hispanic adult cohorts [37]. Admittedly, our sample is considerably smaller and, thus, weak effects may have remained undetected. However, according to our Bayesian analysis, rs174570 (*FADS2*) is the most implausible of the considered candidates, since, in contrast to the other variants, it is dismissed for all three lipid phenotypes analysed.

For other variants, even large sample-sized and high-powered adult studies gave controversial results. While a significant association of rs6102059 (*MAFB*) with LDL-C was observed by some [8], this could not be replicated by others [15]. However, the cohort of supposed European ancestry consisted of self-identified European Americans. Their genetic origin was not validated, which might have blurred the associations. In our Central European sample we observed no convincing association of rs6102059 with TC and LDL-C levels but we can also not completely rule out an effect based on our Bayesian analysis.

The intronic SNP rs3846663 in *HMGCR* was reported to be significantly associated with LDL-C levels in a cohort of 19,840 subjects [8]. These findings were replicated in several populations (Kosrean islands inhabitants (n = 2,346) with an even larger effect size compared to Kathiresan et al [8] and Japanese [38] or Scottish [39]. Our results did not reach significance again possibly due to our limited sample size, but by trend, are in line with the above mentioned studies. This is further confirmed by our Bayesian analysis complying with a possible causal effect of rs3846663 on LDL-C but not on HDL-C or TG.

Rs3812316 (*MLXIPL*) was most strongly associated with TG in adults [16], although others did not find this association [40, 41]. It was suggested that the effect on TG levels must be weak if it exists at all [40]. In our study, standard linear regression analysis did not reveal any significant association. Nevertheless our results show lower triglyceride levels in homozygous SNP-carriers with rs3812316/GG genotype (CC: 1.13 mmol/l; GG: 0.69 mmol/l adjusted for age, sex and BMI SDS) in agreement with the above mentioned observations. The effects that were seen in our analysis indicate a protective function for minor allele carriers concerning triglyceride levels in children, even in the presence of obesity [16]. Again, Bayesian model analysis supports this finding since in contrast to HDL-C and LDL-C, an effect of rs3812316 to TG cannot be excluded.

A limitation of our study is the relatively small sample size since recruitment of volunteers is more challenging for childhood cohorts. Children are a population much less affected by chronic diseases or medication. Therefore, genetic studies in childhood cohorts are intriguing. Indeed, we were able to confirm the association for children for variants which are originally detected in much larger cohorts of adults comprising several thousands of individuals. Although, the power of our study is limited, we could confirm rs599839 and rs4420638 to be associated in children. Interestingly, higher effect sizes compared to adults were observed. However, one has to note that our study population is mainly obese. Therefore, replication in a population-based sample of children is required to show general validity of our associations.

Also, besides the possibility that due to the lower influence of co-morbidities and other life-style related factors, primary genetic effects may be hypothesized to be stronger in children than in adults, an alternative possibility would be the later emergence of genetic effects on phenotype. This would particularly apply to conditions where genetic predisposition is reinforced by additional (environmental) risk factors that accumulate or increase with life time (double/multiple hit theory). Such a relationship has been discussed for the manifestation of coronary artery disease in patient with genetic risk factors [42]. It also needs to be considered, that children and adolescents do not yet present with overt disease and hence do not meet the pathological endpoints (eg. myocardial infarction), which limits interpretation on genotype-phenotype associations.

For adults it is common practise to combine diverse cohorts (i.e. The Framingham Heart Study, Invecchiare in Chianti, London Life Science Population Cohort [8], The Rotterdam Study [10], Diabetes Genetics Initiative [7, 8] or The Finland-United States Investigation of NIDDM Genetics [8, 9, 16]. These cohorts differ considerably regarding the burden of chronic illness or drug-intake which might lower the chance to detect genetic associations. However, besides all the advantages of childhood cohorts, we have to acknowledge that studies in adolescent individuals might be affected by changes of lipid metabolism during puberty [43].

By our Bayesian modelling approach we propose an innovative method of analysing multi-SNP–multi-phenotype associations independently and in addition to the classical frequentist regression modelling. This type of analysis overcomes a number of limitations of classical regression analysis: First, it allows comparisons of different types of models, i.e. different modes of inheritance and inclusion of co-variables. Although it is possible in principle to include multiple SNPs and covariables in regression analysis, this usually results in a large number of possible models with no generally accepted decision rule how to select an optimal one. Second, it considers polygenic effects and the information of other phenotypes as well. Considering the correlation structure between different phenotypes can improve detection of the underlying signal [29]. To some extent, this also allows inference regarding unconfounded effects of genotypes and co-variables, which may be indicative for direct or even causal relationships. Interestingly, as discussed above, our Bayesian model results are always in line with observations in adult studies and hence support these results.

Third, the Bayesian approach can deal with missing values, i.e. single missings in either phenotypes, co-variables or SNPs [44]. For example a classical analysis of all SNPs and phenotypes in parallel would reduce the sample size from 594 to 521 in our study whereas Bayesian analysis includes all individuals resulting in higher power. Indeed, compared to the classical analysis, standard deviations of effect estimates are typically smaller, i.e. estimates are more precise [30] and may handle smaller sample sizes.

## Summary

We could show for the first time in children that rs599839 (*SORT1*) and rs4420638 (*APOE*) are strongly associated with alterations in blood lipid levels independent of the presence and degree of obesity. Our integrative Bayesian model analysis provided further candidate associations requiring further investigation of the candidates. Therefore, we conclude that this novel approach can improve the detection of weaker associations in genotype-phenotype data sets.

## Supporting Information

S1 Data Excel sheet of raw data: Variable sample_id is the sample identifier. SNP genotype corresponds to the number of minor alleles (0, 1 or 2) and 3 refer to missing values. Lipid phenotype levels are provided as hdl_c (high density lipoprotein cholesterol), ldl_c (low density lipoprotein cholesterol, tc (total cholesterol) and tg (triglyceride). Accordingly, the covariables age, sex and bmi_sds are provided.
(XLSX)

S1 Fig Comparison of standard errors. (DOCX)

S2 Fig Results. Effects of identified genetic risk variants on LDL-C.
(DOCX)

S1 Methods Details of Bayesian modelling. (DOCX)

S2 Methods Derivation of Bayes factors for models. (DOCX)

S3 Methods WinBUGS model for Bayesian model selection. (DOCX)

S1 Results Genetic models for BMI SDS and lipid phenotypes. (DOCX)

S2 Results Estimated covariances. (DOCX)

S1 Table Primer and probes for gene expression analysis. (DOCX)

S2 Table Primer sequences for genotyping. (DOCX)

S3 Table Selection and characteristics of lipid variants. (DOCX)

## Abbreviations

*APOE*: apolipoprotein E
TC: total-cholesterol
*FADS2*: fatty acid desaturase 2
GWA: genome-wide association
*HMGCR*: 3-hydroxy-3-methylglutaryl-Coenzyme A reductase
*MAFB*: V-maf musculoaponeurotic fibrosarcoma oncogene homolog B
*MLXIPL*: *MLX interacting protein*
*SORT1*: sortilin 1
