# Supplementary material for: Genetic Contribution of Variants near SORT1 and APOE on LDL Cholesterol Independent of Obesity in Children
Source: PLoS One. 2015 Sep 16;10(9):e0138064. doi: 10.1371/journal.pone.0138064 (PMC4573320; doi:10.1371/journal.pone.0138064)
Supplement: S1 Fig — (DOCX) [file pone.0138064.s002.docx]

# S8 Figure Comparison of standard errors.

Comparison of standard errors of the classical analysis and corresponding standard errors (actual standard deviations of effect estimates) of the Bayesian analysis. Depicted are 36 standard errors originating from analysis of dominant and recessive effects of six SNPs (rs599839, rs3846663, rs3812316, rs174570, rs4420638, rs6102059) tested with three lipid phenotypes (HDL-C, LDL-C, TG). The linear model of the classical analysis consists either of the dominant or the recessive part of the SNP and is adjusted for age, sex and BMI SDS. For Bayesian model analysis, standard errors are averaged over all models containing the respective dominant or recessive covariable. The grey line indicates unity. Altogether 30 out of 36 standard errors of the Bayesian analysis are smaller than the corresponding standard errors of the classical analysis.
