# Supplementary material for: Genetic Contribution of Variants near SORT1 and APOE on LDL Cholesterol Independent of Obesity in Children
Source: PLoS One. 2015 Sep 16;10(9):e0138064. doi: 10.1371/journal.pone.0138064 (PMC4573320; doi:10.1371/journal.pone.0138064)
Supplement: S2 Fig — Effects of identified genetic risk variants on LDL-C. (DOCX) [file pone.0138064.s003.docx]

**S10 Effects of identified genetic risk variants on LDL-C.**

We present the most probable poly-genetic model for LDL-C obtained by Bayesian model analysis. Only recessive effects of rs4420638 and rs599839 were detected. We define a genetic risk score of increased LDL-C by counting the number of genetic variants associated with higher LDL-C: According to the identified genetic model, the risk variants are one/two copies of the minor allele of rs4420638 and two copies of the major allele of rs599839.

The score is zero if an individual lacks both variants. It is one if either the first or the second variant is present. The score is two if both variants are present. We present LDL-C levels in dependence on the genetic risk score. LDL-C is adjusted for age, sex and BMI SDS.
