# Supplementary material for: Genetic Contribution of Variants near SORT1 and APOE on LDL Cholesterol Independent of Obesity in Children
Source: PLoS One. 2015 Sep 16;10(9):e0138064. doi: 10.1371/journal.pone.0138064 (PMC4573320; doi:10.1371/journal.pone.0138064)
Supplement: S1 Methods — (DOCX) [file pone.0138064.s004.docx]

## **S4 Details of Bayesian Modelling.**

The major drawback of the classical analysis is the large number of tests to be performed due to the large number of possible combinations of SNPs and phenotypes and the uncertainty regarding the genetic model. In classical analysis, one has to define a certain model a priori and perform testing thereafter under the assumption that the model is true. Moreover, information of SNPs, covariables and phenotypes not considered in the specific model are ignored resulting in a restricted view on the data. To overcome these limitations, we performed Bayesian model analysis in addition to our classical association analysis. By this approach, we can estimate plausibilities of different models and sizes of genetic and non-genetic effects considering all available information.

Similar to the univariate analysis, transformed and standardised data were used. Lipid parameters were modelled with the Bayesian variable selection approach described in^[[1]](#footnote-1),^^[[2]](#footnote-2)^ using the reversible jump interface of WinBUGS (Version 1.4.3). Since correlation of TC and LDL-C is very high (r=0.91) we studied a model of the (three-dimensional) lipid phenotype HDL-C, LDL-C and TG. We aimed to identify the most plausible sets of co-variables explaining each lipid parameter under consideration of correlations between them.

In our analysis, the set of co-variables consists of age, BMI SDS, sex and a recessive and a dominant part for each of the six SNPs. Decomposing SNPs into a recessive and dominant part of the major allele allows for biological interpretation of genetic effects^[[3]](#footnote-3)^. This is done by defining indicator variables “genotype”=0 and “genotype”=2 for the recessive and dominant part of the SNP’s major allele, respectively. If only one of both SNP indicator variables is selected, the influence is either dominant or recessive. If both indicator variables are included, different levels of co-dominance can be expressed by corresponding effect estimates. Altogether, 15 co-variables were available for selection for each of the 3 lipid parameters. Each different subset of these co-variables forms a model, resulting in a total of 32,768 possible models per (one-dimensional) lipid phenotype. Thus, without preferences, each model has a priori probability of 0.0031%. Given these probabilities and our data, Bayesian posterior probabilities can be estimated measuring the plausibilities of possible models. In consequence, rather than selecting a single model, we assign probabilities for all possible models.

Effect estimates of co-variables can be determined in the Bayesian context by averaging over all models containing this co-variable (Bayesian model averaging) weighted by the plausibility of the model. Results can be considered as analogons to Beta-coefficients of classical linear regression analysis.

The Bayesian model was fitted using 60,000 iterations and two Markov chains. We discarded the first 10,000 iterations (burn-in) of each chain and analysed the samples of the remaining 100,000 iterations. Since convergence of both chains is an important issue, we used “over-dispersed” starting values and compared the results of both chains as described elsewhere^[[4]](#footnote-4)^.

1. Lunn, D. J., Whittaker, J. C. & Best, N. A Bayesian toolkit for genetic association studies. *Genet. Epidemiol.* **30**, 231–247 (2006). [↑](#footnote-ref-1)
2. Lunn, D. J., Best, N. & Whittaker, J. C. Generic reversible jump MCMC using graphical models. Stat Comput 19, 395–408 (2009). [↑](#footnote-ref-2)
3. Lunn, D. J., Whittaker, J. C. & Best, N. A Bayesian toolkit for genetic association studies. *Genet. Epidemiol.* **30**, 231–247 (2006). [↑](#footnote-ref-3)
4. Lunn, D. J. Automated covariate selection and Bayesian model averaging in population PK/PD models. *J Pharmacokinet Pharmacodyn* **35**, 85–100 (2008). [↑](#footnote-ref-4)
