# Supplementary material for: Genetic Contribution of Variants near SORT1 and APOE on LDL Cholesterol Independent of Obesity in Children
Source: PLoS One. 2015 Sep 16;10(9):e0138064. doi: 10.1371/journal.pone.0138064 (PMC4573320; doi:10.1371/journal.pone.0138064)
Supplement: S2 Methods — (DOCX) [file pone.0138064.s005.docx]

# 5 Derivation of Bayes factors for models.

Bayes factors[[1]](#footnote-1) are useful for interpretation of Bayesian model results. They measure the gain in plausibility regarding a specific hypothesis given the data. For example, considering the above mentioned prior probability of a model of about 0.0031% and deriving a posterior probability of the same model of about 3.08% (such as for the model BMI SDS + age for HDL-C) corresponds to a Bayes factor of 1041. Note that the Bayes factor is not the quotient of the two probabilities but the odds-ratio.

We now explain the calculation of Bayes factors in more detail: For simplification, assume an univariate normal response $Y\sim N(\mu,\sigma^{2})$ with observations y_i_, i=1,...,n, individual means µ_i_ and variance $\sigma^{2}$. In our context, variable selection refers to assignment of co-variables x_ij_, i=1,...,n, j=1,...,c to the distribution mean of each observation $\mu_{i}=(1,x_{i\theta_{1}},x_{i\theta_{2}},...,x_{i\theta_{k}})\mathbf{\beta}$ with vector $\mathbf{\theta}=(\theta_{1},\theta_{2},...,\theta_{k})^{T}$ of k column indices corresponding to the selected x_ij_ and $\mathbf{\beta}=(\beta_{1},\beta_{2},...,\beta_{k+1})^{T}$ of k+1 regression coefficients.

The prior for all models with dimension k is prescribed by $P(\theta|k)=\left(\begin{matrix} c \\ k \end{matrix}\right)^{-1}$, i.e. each model is equally probable. If one chooses the prior k~Bin(0.5,c) for model dimension, then all possible models for µ_i_ are equally likely a priori, because $P(\theta,k)=P(\theta|k)P(k)=\left(\begin{matrix} c \\ k \end{matrix}\right)^{-1}\left(\begin{matrix} c \\ k \end{matrix}\right)\left(\frac{1}{2}\right)^{k}\left(\frac{1}{2}\right)^{c-k}=\frac{1}{2^{c}}$.

If Y is a multivariate normal response variable, variable selection is done for each of the distribution means separately as described above. More details of the variable selection approach can be found elsewhere[[2]](#footnote-2).

A Bayes factor is defined as the ratio of the posterior odds to the prior odds. For models, odds is the ratio of probabilities for choosing a certain model m to choosing any other model. Let P(m|Y) the posterior probability of m after observation of Y and $P(m)=\frac{1}{2^{c}}$ the prior probability, then the Bayes factor for m is calculated as $BF(m)=\frac{\frac{P(m|Y)}{1-P(m|Y)}}{\frac{P(m)}{1-P(m)}}=\frac{P(m|Y)}{1-P(m|Y)}\left(2^{c}-1\right)$.

1. Kass, R. E. & Raftery, A. E. Bayes Factors. *Journal of the American Statistical Association* **90**, 773–795 (1995). [↑](#footnote-ref-1)
2. Lunn, D. J., Whittaker, J. C. & Best, N. A Bayesian toolkit for genetic association studies. *Genet. Epidemiol.* **30**, 231–247 (2006). [↑](#footnote-ref-2)
