# Supplementary material for: Genetic Contribution of Variants near SORT1 and APOE on LDL Cholesterol Independent of Obesity in Children
Source: PLoS One. 2015 Sep 16;10(9):e0138064. doi: 10.1371/journal.pone.0138064 (PMC4573320; doi:10.1371/journal.pone.0138064)
Supplement: S3 Methods — (DOCX) [file pone.0138064.s006.docx]

# S7 WinBUGS model for Bayesian model selection.

01 model {

02

03 for (i in 1:n) {

04 y[i,1:p] ~ dmnorm(y.m[i,1:p],y.t[1:p,1:p])

05

06 for (j in 1:q) {

07 x[i,2*j-1]<-equals(g[i,j],0)

08 x[i,2*j]<-equals(g[i,j],2)

09 g[i,j] ~ dgene.aux(psi[i,j],psj[i,j])

10 }

11

12 psi[i,1:q] ~ dmnorm(p.m[1:q],p.t[1:q,1:q])

13 psj[i,1:q] ~ dmnorm(p.m[1:q],p.t[1:q,1:q])

14

15 for (j in 1:r) { x[i,j+2*q]<-g[i,j+q] }

16 }

17

18 for (t in 1:p) {

19

20 for (i in 1:n) { y.m[i,t]<-y.j[t,i] }

21

22 y.j[t,1:n]<-jump.lin.pred(x[1:n,1:c],k[t],b.t)

23 y.i[t]<-jump.model.id(y.j[t,1:n])

24 y.p[t,1:(c+1)]<-jump.lin.pred.pred(y.j[t,1:n],x.p[1:(c+1),1:c])

25

26 for (i in 1:c) {

27 e[t,i]<-y.p[t,i]-y.p[t,c+1]

28 w[t,i]<-step(abs(e[t,i])-del)

29 }

30

31 k[t] ~ dbin(.5,c)

32 }

33

34 y.t[1:p,1:p] ~ dwish(iii[1:p,1:p],p)

35 y.s[1:p,1:p]<-inverse(y.t[1:p,1:p])

36

37 for (i in 1:(c+1)) { for (j in 1:c) { x.p[i,j]<-equals(i,j) } }

38

39 p.m[1:q] ~ dmnorm(p.a[1:q],p.b[1:q,1:q])

40 p.t[1:q,1:q] ~ dwish(p.c[1:q,1:q],q)

41

42 for (i in 1:q) {

43 for(j in 1:q) {

44 p.b[i,j]<-eps*equals(i,j)

45 p.c[i,j]<-q*equals(i,j)

46 }

47 p.a[i]<-0

48 }

49 }

Constants are defined in the data section outside the model definition above and are explained below, e.g. “n”=594 is the number of observations and “p”=3 is the number of distribution means estimated by variable selection and refers to the 3 lipid phenotypes. HDL-C, LDL-C and TG data are given as “y[,1:3]”. The distributions means “y.m[,1:p]” (line 4) depend on the selected co-variables for each of the 3 lipids. The corresponding precision matrix (inverse covariance matrix) “y.t” is defined by a Wishart prior (line 34). Further, “iii” is the 3 by 3 identical matrix and “y.s” is the corresponding covariance matrix (line 35). Constants “q”=6 and “r”=3 refer to number of SNPs and to the number of additional co-variables age, BMI SDS and sex, respectively. Genotype data is given as “g[,1:6]” and age, BMI SDS and sex as “g[,7:9]”. The content of “x[,1:15]” is used for variable selection and is constructed from “g[,1:9]” (lines 7-8,15). Matrix “x[,1:12]” consists of indicator variables for the recessive and dominant part of each SNP and “x[,13:15]“ contains age, BMI SDS and sex. For inferring missing genotypes (line 9), two pseudo-haplotypes are estimated (lines 12-13) and appropriate priors defined (lines 39-48), where “eps”=1e-4. Individual means are predicted by a function call (line 22) and are filled in the matrix of means “y.j”. Constant “b.t”=1e-4 is the prior precision of all effect estimates. Variables “k” relate to the number of selected co-variables and are defined with binomial prior (line 31). Transposing “y.j” yields “y.m” (line 20). Constant “c”=15 corresponds to the total number of co-variables available for selection (“c=2*q+r”). Each model is identified by “y.i” (line 23). Effect estimates “e” are calculated (line 27) by predicting the response “y.p” (line 24) for a certain design matrix “x.p” (line 37). The (marginal) inclusion of a co-variable in any model is indicated by “w” and equals 1 if the absolute value of “e” exceeds a small constant “del”=1e-20 (line 28). More detailed explanations on the basis of a similar example can be found elsewhere^[[1]](#footnote-1)^.

1. Lunn, D. J., Whittaker, J. C. & Best, N. A Bayesian toolkit for genetic association studies. *Genet. Epidemiol.* **30**, 231–247 (2006). [↑](#footnote-ref-1)
