# Supplementary material for: Genetic Contribution of Variants near SORT1 and APOE on LDL Cholesterol Independent of Obesity in Children
Source: PLoS One. 2015 Sep 16;10(9):e0138064. doi: 10.1371/journal.pone.0138064 (PMC4573320; doi:10.1371/journal.pone.0138064)
Supplement: S1 Results — (DOCX) [file pone.0138064.s007.docx]

# S6 Genetic models for BMI SDS and lipid phenotypes.

# We present case numbers (N), beta estimates of standard linear regression analysis and corresponding standard errors (SE) and p-values for all three genetic models (additive, dominant, recessive) analysed. Since standardized values were analysed, beta estimates and standard errors have unit 1. BMI SDS was adjusted for age and sex. Lipid phenotypes were adjusted for age, sex and BMI SDS. Lines with significant associations are printed in bold type.

| Phenotype | Variant | N | Additive Beta | Additive SE | Additive p-value | Dominant Beta | Dominant SE | Dominant p-value | Recessive Beta | Recessive SE | Recessive p-value |
| --- | --- | --- | --- | --- | --- | --- | --- | --- | --- | --- | --- |
| BMI SDS | rs599839 | 576 | -0.0866 | 0.0664 | 0.1926 | -0.2365 | 0.1693 | 0.1631 | 0.0764 | 0.0819 | 0.3512 |
| BMI SDS | rs3846663 | 572 | 0.0756 | 0.0614 | 0.2187 | 0.1966 | 0.1251 | 0.1165 | -0.0529 | 0.0841 | 0.5291 |
| BMI SDS | rs3812316 | 564 | -0.1261 | 0.0948 | 0.1837 | -0.17 | 0.4107 | 0.6791 | 0.1359 | 0.1021 | 0.1838 |
| BMI SDS | rs174570 | 578 | 0.1761 | 0.0903 | 0.0516 | 0.1779 | 0.3529 | 0.6144 | -0.1979 | 0.099 | 0.0462 |
| BMI SDS | rs4420638 | 584 | -0.0041 | 0.0793 | 0.9585 | -0.0561 | 0.2782 | 0.8403 | -0.0005 | 0.0883 | 0.9953 |
| BMI SDS | rs6102059 | 575 | 0.0625 | 0.0648 | 0.3353 | 0.2454 | 0.1496 | 0.1014 | -0.027 | 0.0829 | 0.7443 |
| **TC** | **rs599839** | **576** | **-0.2565** | **0.0672** | **1.50e-04** | **-0.2766** | **0.1733** | **0.111** | **0.325** | **0.0828** | **9.67e-05** |
| TC | rs3846663 | 572 | 0.1413 | 0.0622 | 0.0235 | 0.2657 | 0.127 | 0.0369 | -0.1445 | 0.0852 | 0.0904 |
| TC | rs3812316 | 564 | -0.0224 | 0.094 | 0.8118 | -0.3501 | 0.4059 | 0.3888 | 0.0042 | 0.1013 | 0.9667 |
| TC | rs174570 | 578 | -0.0401 | 0.0917 | 0.6625 | -0.295 | 0.3561 | 0.4078 | 0.0247 | 0.1007 | 0.8063 |
| **TC** | **rs4420638** | **584** | **0.336** | **0.079** | **2.45e-05** | **0.5642** | **0.2805** | **0.0448** | **-0.3597** | **0.0881** | **5.02e-05** |
| TC | rs6102059 | 575 | 0.0185 | 0.0649 | 0.7753 | 0.3292 | 0.1497 | 0.0283 | 0.0699 | 0.0829 | 0.3992 |
| HDL-C | rs599839 | 576 | 0.0766 | 0.0665 | 0.2499 | 0.28 | 0.1696 | 0.0993 | -0.0511 | 0.0821 | 0.5339 |
| HDL-C | rs3846663 | 572 | 0.0981 | 0.0607 | 0.1064 | -0.007 | 0.1241 | 0.9549 | -0.1863 | 0.0827 | 0.0247 |
| HDL-C | rs3812316 | 564 | 0.1287 | 0.0932 | 0.1677 | 0.3968 | 0.4029 | 0.325 | -0.1247 | 0.1004 | 0.2148 |
| HDL-C | rs174570 | 578 | -0.0775 | 0.0896 | 0.3873 | -0.0765 | 0.3483 | 0.8262 | 0.0872 | 0.0983 | 0.3754 |
| HDL-C | rs4420638 | 584 | -0.1297 | 0.0782 | 0.098 | -0.4498 | 0.2746 | 0.1019 | 0.1154 | 0.0872 | 0.186 |
| HDL-C | rs6102059 | 575 | 0.0384 | 0.0637 | 0.5474 | 0.0655 | 0.1476 | 0.6574 | -0.0427 | 0.0814 | 0.6004 |
| **LDL-C** | **rs599839** | **576** | **-0.2996** | **0.0668** | **8.82e-06** | **-0.4266** | **0.1725** | **0.0137** | **0.3555** | **0.0824** | **1.90e-05** |
| LDL-C | rs3846663 | 572 | 0.1196 | 0.0619 | 0.0539 | 0.3028 | 0.1262 | 0.0167 | -0.0875 | 0.0848 | 0.3025 |
| LDL-C | rs3812316 | 564 | -0.0425 | 0.0935 | 0.6494 | -0.1414 | 0.4042 | 0.7267 | 0.0406 | 0.1008 | 0.6872 |
| LDL-C | rs174570 | 578 | 0.0212 | 0.0913 | 0.8165 | -0.2008 | 0.3545 | 0.5714 | -0.0415 | 0.1002 | 0.6785 |
| **LDL-C** | **rs4420638** | **584** | **0.3819** | **0.0783** | **1.38e-06** | **0.6728** | **0.2789** | **0.0161** | **-0.4057** | **0.0873** | **4.18e-06** |
| LDL-C | rs6102059 | 575 | 0.018 | 0.0648 | 0.7811 | 0.3394 | 0.1493 | 0.0233 | 0.0739 | 0.0827 | 0.3715 |
| TG | rs599839 | 576 | -0.1135 | 0.065 | 0.0811 | -0.2432 | 0.1659 | 0.1433 | 0.1157 | 0.0801 | 0.1493 |
| TG | rs3846663 | 572 | -0.0065 | 0.0593 | 0.913 | 0.0888 | 0.1209 | 0.4631 | 0.0519 | 0.081 | 0.5218 |
| TG | rs3812316 | 564 | -0.1342 | 0.0878 | 0.1269 | -0.8484 | 0.3784 | 0.0254 | 0.103 | 0.0947 | 0.277 |
| TG | rs174570 | 578 | 0.1367 | 0.0868 | 0.1159 | 0.177 | 0.3378 | 0.6005 | -0.1504 | 0.0953 | 0.1148 |
| TG | rs4420638 | 584 | 0.1352 | 0.076 | 0.0758 | 0.3752 | 0.2671 | 0.1607 | -0.1299 | 0.0847 | 0.1258 |
| TG | rs6102059 | 575 | 0.0754 | 0.062 | 0.2245 | 0.1639 | 0.1436 | 0.2544 | -0.0732 | 0.0793 | 0.3564 |
