# Supplementary material for: Genetic Contribution of Variants near SORT1 and APOE on LDL Cholesterol Independent of Obesity in Children
Source: PLoS One. 2015 Sep 16;10(9):e0138064. doi: 10.1371/journal.pone.0138064 (PMC4573320; doi:10.1371/journal.pone.0138064)
Supplement: S2 Results — (DOCX) [file pone.0138064.s008.docx]

# S9 Estimated covariances.

The estimated covariance matrix for $\left(HDL-C,LDL-C,TG\right)\sim MVN_{3}\left(\mathbf{\mu},\mathbf{\Sigma}\right)$ is $\hat{\mathbf{\Sigma}}=\left(\begin{matrix} 0.9619 & 0.0283 & \text{-0.3352} \\ 0.0283 & 0.9417 & 0.3063 \\ \text{-0.3352} & 0.3063 & 0.9060 \end{matrix}\right)$
