# Supplementary material for: Genetic Contribution of Variants near SORT1 and APOE on LDL Cholesterol Independent of Obesity in Children
Source: PLoS One. 2015 Sep 16;10(9):e0138064. doi: 10.1371/journal.pone.0138064 (PMC4573320; doi:10.1371/journal.pone.0138064)
Supplement: S1 Table — (DOCX) [file pone.0138064.s009.docx]

# S1 Table. Primer and probes for gene expression analysis

| Gene | Forward primer | Reverse Primer | Probe | Template length (bp) |
| --- | --- | --- | --- | --- |
| APOE | GCACTGGGTCGCTTTTGG | TGTAGGCCTTCAACTCCTTCATG | CGCCCTCAGTTCCTGGGTGACCT | 139 |
| FADS2 | GAACATGCTGCACGTGTTTGT | GGGCAGGTATTTCAGCTTCTTC | TGGCAGCCCATCGAGTACGGC | 76 |
| HMGCR | TTGTCACCGCCATCTACATTG | GGGACCACTTGCTTCCATTAAAG | ACAGGATGCAGCACAGAATGTTGGTAGTTCA | 78 |
| MAFB | CATCTGGAGACTCCTGGCTTTCT | TCCTGGCGCGGACTACTCT | TGTCCCGGCTTAACGCGCAAAGT | 84 |
| MLXIPL | CCCACACTCACACGCCTCTT | GAAATTCTTCCACTTGGGAGACA | GCCACTGTAGGCCAGGCTCAAGCACT | 98 |
| SORT1 | CGCACCAGCATGTGTTTGAT | AGACTAGAATGACCCCAGTGCTATCT | CTCAGAGGCTCAGTATCCTTGTCCTGGGT | 81 |
| TBP | TTGTAAACTTGACCTAAAGACCATTGC | TTCGTGGCTCTCTTATCCTCATG | AACGCCGAATATAATCCCAAGCGGTTTG | 101 |
| USF2 | GCGTCCAGTGTGGGAGATACTAC | GGCGATCGTCCTCTGTGTTC | AGAGCTTGCAGGCTGGAGGCCAGTT | 132 |
| HPRT | GGCAGTATAATCCAAAGATGGTCAA | GTCTGGCTTATATCCAACACTTCGT | CAAGCTTGCTGGTGAAAAGGACCCC | 80 |
