# Supplementary material for: Genetic Contribution of Variants near SORT1 and APOE on LDL Cholesterol Independent of Obesity in Children
Source: PLoS One. 2015 Sep 16;10(9):e0138064. doi: 10.1371/journal.pone.0138064 (PMC4573320; doi:10.1371/journal.pone.0138064)
Supplement: S2 Table — (DOCX) [file pone.0138064.s010.docx]

# S2 Table. Primer sequences for genotyping

| Gene | NCBI SNP ID | SNP type / localisation | Assay ID  TaqMan(R) Genotyping Assays | Context Sequence/Primer and Probe Sequence  ……[VIC/FAM]……. | Rate of concordance |
| --- | --- | --- | --- | --- | --- |
| MAFB | rs6102059 | INTERGENIC/UNKNOWN?  LOC102724968/ XR_430319.1  ~85 kBp at 3' side of MAFB | C__30612159_10 | CCTCTCAACAGCCCTCTGATCTACG[C/T]ACACTAACATCATGCCCATTTTACA | 100%  (90/90) |
| APOE | rs4420638 | INTERGENIC of APOC1 and  APOC1P1  ~10 kBp at 3' side of APOE | AHBJDXZ  Custom SNP Taqman Assay | APOEC1C2C-APEM2: 5’-6FAM-CCACACCAGGAAA-MGBNFQ-3’  APOEC1C2C4-APOEV2: 5’-VIC-TAGACCACACTAGGAAAA-MGBNFQ-3’  APOEC1C2C4-APOEF: TCAGCCTAGCAATGTCACTATGC  APOECC2C4-APOER: AAAAACCTCAGCCCCTCATCTC | 100%  (93/93) |
| SORT1 | rs599839 | INTERGENIC of PSRC1 and CELSR2  ~10 kBp at 3' side of SORT1 | C____972962_10 | AAGAGAAAGAAATAGGAGCAGGATC[A/G]ACTTCCAGATATACAGAGAATATAA | 100%  (91/91) |
| HMGCR | rs3846663 | penultimate intron of HMGCR | C__26555315_10 | GTCTAGTTCTATTCTGATGCCATTA[C/T]AGTTGCCCTGTTTTTAGTTGATTTA | 100%  (130/130) |
| FADS2 | rs174570 | second intron of FADS2 | C___2268960_10 | AGGCAGAAAGGAGGGATGAACTTGA[C/T]GTAGATCATTCCACCTGGAGGTCTA | 100%  (88/88) |
| MLXIPL | rs3812316 | sixth exon of MLXIPL  MIS-SENSE MUTATION  Q241H | C___2632498_10 | ACAAAAAGCAATTGAGGTCCAGGAG[C/G]TGCCGCCCACCCGGCTCCTCCTCTG | 100%  (104/104) |
