# Supplementary material for: Genetic Contribution of Variants near SORT1 and APOE on LDL Cholesterol Independent of Obesity in Children
Source: PLoS One. 2015 Sep 16;10(9):e0138064. doi: 10.1371/journal.pone.0138064 (PMC4573320; doi:10.1371/journal.pone.0138064)
Supplement: S3 Table — (DOCX) [file pone.0138064.s011.docx]

**S3 Table Selection and characteristics of lipid variants**.

| Variant | rs599839 | rs3846663 | rs3812316 | rs174570 | rs4420638 | rs6102059 |
| --- | --- | --- | --- | --- | --- | --- |
| Chromosome | 1 | 5 | 7 | 11 | 19 | 20 |
| Position | 109623689 | 74691482 | 72658273 | 61353788 | 50114786 | 38662198 |
| Alleles (major/minor) | A/G | C/T | C/G | C/T | A/G | C/T |
| Gene | *SORT1* | *HMGCR* | *MLXIPL* | *FADS2* | *APOE* | *MAFB* |
| Product | sortilin 1 | 3-hydroxy-3-methylglutaryl-Coenzyme A reductase | MLX interacting protein | fatty acid desaturase | apoE | V-maf musculoaponeurotic fibrosarcoma oncogene homolog B |
| Function | co-receptor for neuropeptides | Enzyme chol. Synthesis | TF in TG synthesis | Enzyme FA metabolism | apolipoprotein, chol. Transport | TF in hematopoeisis |
| Trait | LDL-C | LDL-C | TG | TG, TC | LDL-C | LDL-C |
| Location | intergenic | intronic | Gln>His | intronic | 5´upstream | intergenic |
| N | 576 | 572 | 564 | 578 | 584 | 575 |
| Call rate | 97.0 | 96.3 | 94.9 | 97.3 | 98.3 | 96.8 |
| MAF | 24.1 | 35.8 | 11.3 | 11.7 | 16.8 | 29.7 |
| CEU MAF | 27.9 | 39.4 | 14.2 | 13.4 | 18.3 | 28.3 |
| HWE | 0.73 | 0.72 | 0.83 | 1.00 | 0.37 | 0.69 |

# SNP chromosome, position and CEU MAF were taken from Hapmap release #28,NCBI 36, dbSNP 126. For rs3812316 CEU MAF is obtained from pilot 1 CEU low coverage panel. All genes are on the complement strand. Allele notation refers to the DNA forward strand. Call rate and MAF are given in percent, HWE indicates the *p*-value of the exact HWE-test.
